# Interface Chemical Modification between All-Inorganic Perovskite Nanocrystals and Porous Silica Microspheres for Composite Materials with Improved Emission

**DOI:** 10.3390/nano11010119

**Published:** 2021-01-07

**Authors:** Sergei Cherevkov, Ruslan Azizov, Anastasiia Sokolova, Valeriia Nautran, Mikhail Miruschenko, Irina Arefina, Mikhail Baranov, Dmitry Kurdyukov, Ekaterina Stovpiaga, Valery Golubev, Alexander Baranov, Elena Ushakova

**Affiliations:** 1 Center of Information Optical Technologies, ITMO University, 197101 Saint Petersburg, Russia; s.cherevkov@gmail.com (S.C.); azizov.ruslan.2008@mail.ru (R.A.); physasv@yandex.ru (A.S.); l.nautran@mail.ru (V.N.); miruschenko98mail@yandex.ru (M.M.); irina-arefina97@mail.ru (I.A.); mbaranov@mail.ru (M.B.); a_v_baranov@yahoo.com (A.B.); 2 Laboratory of Amorphous Semiconductor Physics, Ioffe Institute, 194021 Saint Petersburg, Russia; kurd.gvg@mail.ioffe.ru (D.K.); kattrof@gvg.ioffe.ru (E.S.); golubev.gvg@mail.ioffe.ru (V.G.); 3 Department of Materials Science and Engineering and Center for Functional Photonics (CFP), City University of Hong Kong, Hong Kong, China

**Keywords:** lead bromide perovskite, nanocrystals, porous silica, microspheres, photoluminescence

## Abstract

In recent years, there has been rapid progress in the development of photonic devices based on lead halide perovskite nanocrystals since they possess a set of unique optical and charge transport properties. However, the main limiting factor for their subsequent application is poor stability against exposure to adverse environmental conditions. In this work, a study of a composite material based on perovskite CsPbBr_3_ nanocrystals embedded in porous silica microspheres is presented. We developed two different approaches to change the interface between nanocrystals and the surface of the microsphere pores: surface treatment of (i) nanocrystals or (ii) microspheres. The surface modification with tetraethylorthosilicate molecules not only increased stability but also improved the optical responses of the composite material. The position of the emission band remained almost unchanged, but its lifetime increased significantly compared to the initial value. The improvement of the optical performance via surface modification with tetraethylorthosilicate molecules also works for the lead-free Bi-doped Cs_2_AgInCl_6_ double perovskite nanocrystals leading to increased stability of their optical responses at ambient conditions. These results clearly demonstrate the advantage of a composite material that can be used in novel photonic devices with improved performance.

## 1. Introduction

Currently, metal halide perovskite nanocrystals (pNCs) are one of the most promising luminescent nanoparticles since they possess high charge mobility and remarkably high photoluminescence (PL) quantum yields [1,2,3]. Although the pNCs can be synthesized by a variety of different facile methods by means of colloidal chemistry, their crystal structure and hence physicochemical properties may undergo drastic changes upon storage at ambient conditions. There are several ways to solve this problem: passivation of the pNC surface via chemical treatment [4] and embedding the pNCs into inert solid porous matrices are among them [5,6]. The latter method is attractive since it can be applied to all types of pNCs and their mixtures with other nanoparticles including magnetic and plasmonic. A well-chosen matrix not only increases the stability of the pNC, but also improves the luminescent properties of embedded nanoparticles. The use of various synthesis strategies has led to a variety of possible structures of the resulting composite nanostructured materials. pNCs can be placed on the outer shell of silica (SiO_2_) spheres via bonding to ligands [7]; a hollow matrix can be filled with pNCs [8]; silica layer can be grown on the pNC leading to the formation of core-shell structure [9]. Another way to fabricate composites is embedding of pNCs into a porous inert matrix is the straightforward and widely used method to achieve perovskite-based composites with stable performance [6,10,11]. The formation of a double shell, including organic-inorganic layers, almost completely protects the pNCs from the influence of the external environment [12,13]. It is also worth mentioning that even with relatively thin outer inorganic layer (silica) with a thickness of 3–4 nm the passivation of the pNC surface and their protection from the environment are still efficient [14,15].

Tetraethylorthosilicate (TEOS) is widely used as a precursor for the silica matrices formation together with the passivation of pNCs. Related studies showed that TEOS molecules may be used for “during”- and “post“-synthesis treatment of pNCs. By adding TEOS in the reaction flask during the hot-injection pNCs synthesis, a thin shell of silica can be formed at the pNCs “in situ” which prevents the anionic exchange of various halide elements between perovskite pNCs [16,17]. However, during the silica formation, TEOS undergoes hydrolysis which affects the stability of perovskite crystal structure and may lead to complete decomposition. It was shown that by introducing a highly branched clogging ligand, trioctylphosphineoxide, in the reaction mixture the pNCs decomposition can be blocked [18]. Amine-containing molecules, i.e., 3-aminopropyltriethoxysilane (APTES), can be also used as a buffer layer between pNCs and silica formed by TEOS or as both passivation agent and a precursor for silica shell [19]. Although TEOS and APTES molecules proved to be promising ligands for the fabrication of stable pNCs, there is still a lack of information on the optical properties of the nanostructured material based on these modified pNCs, including the influence of the chemical interface on the emission of pNC-based composites.

Indeed, the advantages of using a protective matrix in pNC-based nanostructured materials include not only the preservation of optical properties or even improving pNC emissions but also open a possibility to fabricate active materials for novel photonic devices. For latter applications, matrices should meet the following demands: transparency, stability while the operation of the device, ease of fabrication, and possibility for scaling the production. From the view of lasing applications, the control of the shape of the host matrix in order to vary the shape from fibers to spheres meeting the resonance conditions for further observation of amplified spontaneous emission (ASE) and lasing is needed [20,21]. In recent work [22], it was shown that the fibers based on composites made with pNCs and polymers (polymethyl methacrylate and poly(vinylidene fluoride)) had stable optical responses at humidity at 60% and performed ASE and waveguide lasing. He et al. presented the fabrication of SiN nanoscale cavity covered by pNCs mixed with PMMA which showed lasing with Q-factor of 1.5 × 10^4^ and threshold at 5.62 μJ/cm^2^ [23]. Spherical matrices are implemented to achieve whispering-gallery mode (WGM) lasing [24,25]. Price et al. showed that 9.2 μm silica spheres acted as spherical resonators for all-inorganic pNCs with threshold of 750 μJ/cm^2^ [24]. Yan et al. showed that further improvement of the pNCs surface by 2-hexyldecanoic acid and their further applying to the silica microsphere resulted in WGM lasing with decreased threshold down to 5.47 μJ/cm^2^ [25]. Thus, spherical silica matrices are suitable for fabrication of luminescent composites for lasing applications, including porous spheres that can act not only as WGM resonators but as media for random lasing from the embedded pNCs. However, these systems demand the improvement of the pNC-matrix chemical interface to decrease the lasing threshold and improve Q-factor.

Herein, we showed that treatment of pNCs with TEOS molecules at 80 °C resulted in the preserving optical properties during storage at ambient conditions not only for CsPbBr_3_ pNCs but also for lead-free Bi-doped Cs_2_AgInCl_6_ double pNCs. The performance of pNCs in the composite material can be further preserved and improved by control of the chemical composition of the interface between pNCs and matrix. We developed the formation protocol of composites based on CsPbBr_3_ pNCs and silica microspheres (MSs) which were treated with TEOS molecules or hydrochloric acid (HCl). The modifications of the interface resulted in the improved emission of the composite which is a next step in the development of highly luminescent and stable nanostructured material for photonic applications.

## 2. Materials and Methods

### 2.1. Experimental Setup

Images from scanning transmission electron microscope (STEM) were obtained with scanning electron microscope Merlin (Zeiss, Oberkochen, Germany) at 5 kV and WD = 3.4 mm. Fourier-transform infrared spectra were obtained on a Tenzor II (Bruker, Billerica, MA, USA). Absorption spectral measurements of the samples were performed on a UV-3600 spectrophotometer (Shimadzu, Kyoto, Japan), and luminescent measurements were carried out on a confocal laser scanning microscope LSM-710 (Zeiss, Oberkochen, Germany) equipped with 20× (NA = 0.4) or 50× (NA = 0.95) objectives and a 405 nm laser and on a spectrofluorometer FP-1800 (Jasco, Tokyo, Japan). A confocal microscope MicroTime 100 (Picoquant, Berlin, Germany) equipped with a 100× (NA = 0.95) objective and a 405 nm pulsed diode laser was used to study the PL decay of the samples. For more accurate results, the measurements were performed for several points of the sample. PL decay curves were fitted by a biexponential function: $I\left(t\right)=I_{0}+A_{1}e^{-t/\tau_{1}}+A_{2}e^{-t/\tau_{2}}$. The average PL lifetime has been calculated as $\langle \tau \rangle =\sum A_{i}\tau_{i}{}^{2}/\sum A_{i}\tau_{i}$.

### 2.2. Synthesis

**Figure 1:**
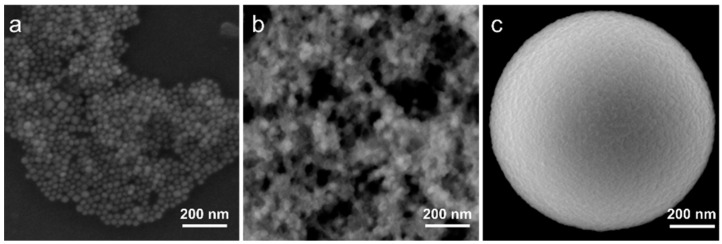
STEM images of (**a**) perovskite nanocrystals (pNCs), (**b**) pNC/tetraethylorthosilicate (TEOS), and (**c**) SEM (secondary electron) image of single microspheres (MSs).

Lead bromide perovskite NCs with chemical formula CsPbBr_3_ were synthesized according to the previously reported protocol [13]. As a result of the synthesis, a colloidal solution of pNCs with a mean size of 18 ± 4 nm was obtained. A typical STEM image is shown in Figure 1a. Since the TEOS molecules are used as precursors for silica MSs, we first prepared the composite with TEOS-treated pNCs to enhance the bonding between the surface of MS’s pores and the surface of pNCs. The sample pNC/TEOS was prepared by post-synthetic treatment of the pNC colloidal solution with an excess of TEOS molecules. For that to 100 µL of stock pNC solution in toluene (10^−5^ M) a 100 µL of TEOS was added, the mixture was stirred over 2 days. Then the pNC/TEOS solution was precipitated by centrifugation, dried at 80 °C in the vacuum oven, and then re-dissolved in 100 µL of toluene. From the STEM image (Figure 1b) it is seen that the pNCs increased in size, thus it can be inferred that the pNCs are covered with TEOS molecules with an average size of pNC/TEOS of 30 ± 10 nm. The scheme of pNC/TEOS preparation is shown in the next section.

Monodisperse MSs of porous silica with a diameter of 1.1 ± 0.1 μm were synthesized according to the previously reported route [26]. The pore volume and size were previously estimated by nitrogen adsorption porosimetry: there are several types of pores with varied pore sizes: ~3 nm and 20–100 nm; the pore volume is ~50%. Typical SEM images of individual MS are shown in Figure 1c and Figure S1 (Supplementary Materials). MSs were first pre-washed as follows: MSs were stirred for 30 s in acetone, sonicated for 30 s, and precipitated by centrifuge at 10,000 rpm for 60 s. To investigate the effect of the interface between MSs and pNCs on the optical properties of the composite, MSs treated with TEOS and HCl were prepared. To 2 mg of MSs, a 200 μL of TEOS or 1 M HCl was added, the mixture was stirred for 30 s, ultrasonicated for 30 s, and left stirring over 2 h. The obtained treated MSs were then washed according to the abovementioned procedure and dried at 100 °C for 1 h in a vacuum oven to remove the residual solvent.

**Figure 2:**
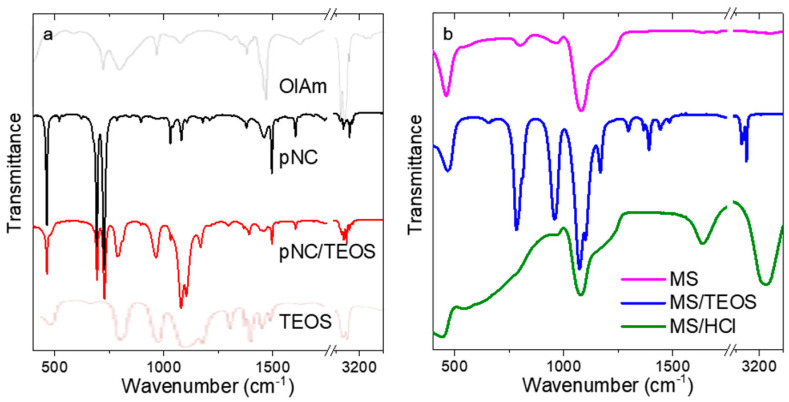
FTIR spectra of: (**a**) initial pNCs (black), pNC/TEOS (red), oleylamine (grey), TEOS (pale brown); (**b**) MSs (magenta), MS/TEOS (blue), and MS/HCl (green).

Before the pNCs embedding into MSs, the chemistry of the surface was examined by means of FTIR spectroscopy (Figure 2). The FTIR spectrum of initial pNCs (Figure 2a) contains peaks at {3033, 2910, 2873, 727} and {1107, 1082} cm^−1^ which are attributed to the –CH and =CH vibrations of oleylamine ligand and to C-N bonding, respectively. It is worth mentioning that the peaks typical for amines (-NH stretching) at 3380–3280 cm^−1^ are absent which shows that the oleylamine ligand is bonded to the pNC surface via a nitrogen atom (Figure 2a, grey line). The pNC/TEOS FTIR spectrum contains peaks at {2910, 2873}, {1166, 1103, 1079}, {965, 790} cm^−1^ which are typical for –CH stretching, Si-O-Si asymmetric stretching and Si-O-C and/or Si-OH bending vibrations of TEOS molecule, respectively [27] (Figure 2a, pale brown line). Thus, it can be inferred that the surface functionalization of pNCs with TEOS molecules underwent successfully. The FTIR spectra of MSs are shown in Figure 2b. The spectrum of bare MSs contains broad peaks at 1210, 1080, and 965 cm^−1^ which are typical for the silica materials and are ascribed to Si-O-Si asymmetric stretching and Si-OH bending vibrations [28]. The TEOS treatment resulted in appearance of the additional peaks in the FTIR spectrum of MS/TEOS sample: {2979, 2935, 2887} and {1485, 1391, 1363, 1296} cm^−1^ which are typical for the TEOS molecule [27]. MS/HCl sample apart from the peaks typical for silica materials shows the wide and intense peaks at 3200 and 1640 cm^−1^ which are typical for polymeric H-bonding and H-OH bending vibration. Thus, these results confirmed the change of the functional groups at the surface of both pNCs and MSs by simple post-synthetic treatment.

## 3. Results and Discussion

**Figure 3:**
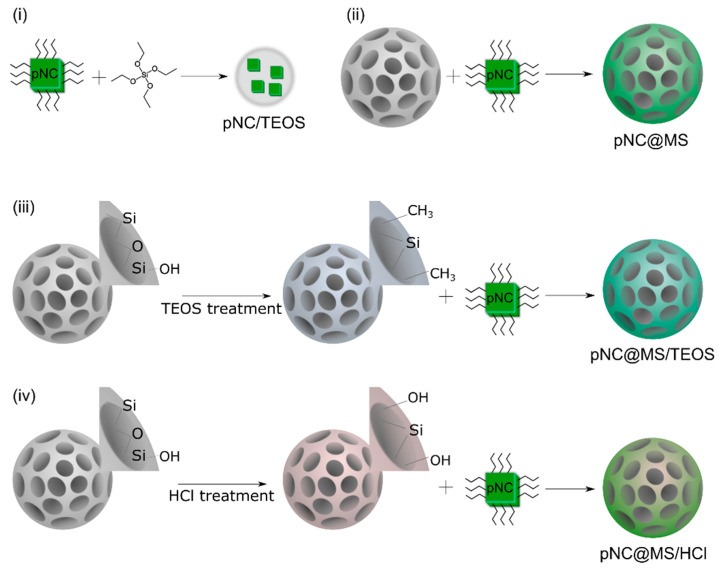
Scheme of the composites’ fabrication. (**i**) pNC/TEOS; (**ii**) pNC@MS; (**iii**) pNC@MS/TEOS; (**iv**) pNC@MS/HCl.

The scheme of pNC/TEOS preparation is shown in Figure 3i. Composite materials based on pNCs and MSs were obtained by embedding pNCs in MSs dispersed in toluene according to Ref. [13]. Briefly, 100 µL pNCs stock solution was added to 2 mg of MSs, stirred by vortex for 30 s, and centrifuged at 2000 rpm for 60 s, followed by removing the supernatant. This step was repeated 3 times. After embedding the pNCs into the MS pores the sample was washed: to the precipitate 500 µL of acetone/octane mixture (1/1 volume ratio) was added to the precipitate, the mixture was stirred by vortex for 30 s and centrifuged at 2000 rpm for 60 s followed by removing the supernatant. The precipitate was dried at 100 C in a vacuum oven for 30 min. Thus, the pNC@MS sample was obtained (Figure 3ii). The pNC@MS/TEOS and pNC@MS/HCl samples were obtained following the abovementioned procedure with treated MSs: MS/TEOS or MS/HCl, respectively (Figure 3iii,iv).

**Figure 4:**
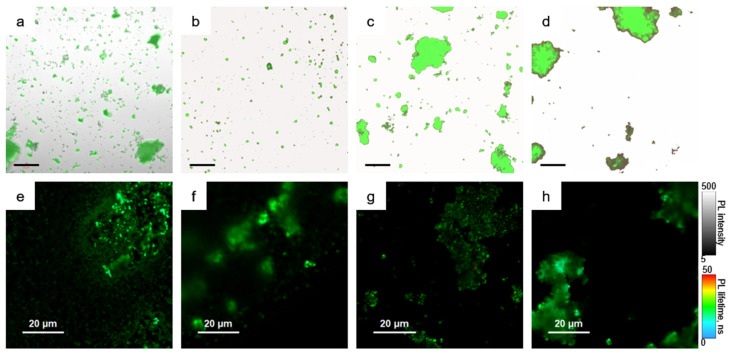
Microscopic images of the samples. (**a**–**d**) combined images from transmission and emission channels, (**e**–**h**) Fluorescence lifetime images. pNC/TEOS (**a**,**e**); pNC@MS (**b**,**f**); pNC@MS/TEOS (**c**,**g**); pNC@MS/HCl (**d**,**h**). Scale in (**a**–**d**) is of 50 μm.

The combination of confocal microscopy and steady-state/time-resolved spectroscopy was used to study morphology related optical responses from the obtained samples. Figure 4 shows the microscopic images of the samples.

From Figure 4a, it is seen that the pNC/TEOS form the agglomerates with preserved pNC emission. The nonemissive agglomerates are formed by TEOS molecules without pNCs. Fluorescence lifetime image of pNC/TEOS (Figure 4e) shows clearly distinguished luminescent particles with the sizes of several hundreds of nm with evenly distributed PL lifetime. From Figure 4b–d it is seen that the percentage of the embedded pNCs into MSs increases in the set pNC@MS/HCl, pNC@MS, and pNC@MS/TEOS.

At the same time, for the samples formed with treated MSs in contrast to the pNC@MS sample, the agglomeration of the MSs is observed which indicates the attractive interaction between treated MSs. At the same time, the fluorescence lifetime images (Figure 4f–h) show that the pNCs in MSs possess evenly distributed PL lifetimes independent from the agglomeration degree of the MSs. We also tried another type of MSs with a smaller diameter (480 nm) and pore volume of 20% with the same pore sizes. The analysis of PL images showed almost the same degree of pNCs embedding with preserving the optical properties of pNCs (Figure S2, Supplementary Materials). This observation shows the opportunity for the development of composite emissive materials based on pNCs and silica microspheres apart from their size which opens a way to align the composite architecture for achieving the conditions for resonance for further lasing applications [20].

**Figure 5:**
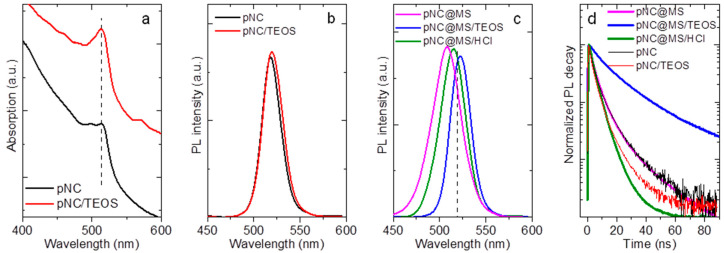
(**a**) Absorption spectra of pNC and pNC/TEOS; photoluminescence (PL) spectra of (**b**) pNC, pNC/TEOS and (**c**) pNC@MS, pNC@MS/TEOS, and pNC@MS/HCl; (**d**) normalized PL decay of pNC, pNC/TEOS, pNC@MS, pNC@MS/TEOS, and pNC@MS/HCl.

Preliminary investigations of the influence of pNC surface chemistry on their optical properties were carried out on the pNCs treated with TEOS and NH_4_Cl molecules. For that pNC colloidal solutions were mixed with saturated solutions of molecules of interest and stirred overnight. The pNCs were then washed thoroughly with acetone to remove the excess of molecules which are not bound to the surface. The absorption and PL spectra (Figure S3, Supplementary Materials) showed that during TEOS molecules treatment the absorption peak position remained the same compared to blueshift observed for pNC solution treated with NH_4_Cl molecules. The PL peak position for TEOS treated pNCs slighlty redshifted by 4 nm and is mainly due to possible agglomeration of particles in solution, while the PL peak position of NH_4_Cl-treated pNCs is blueshifted together with decreased PL intensity. It is caused by partial Br exchange with Cl which is a common phenomenon for perovskite materials resulting in both absorption and PL shift in the blue spectral region [1]. It should be also noted that the full width at half maximum (FWHM) remained almost the same (23–24 nm) for treated pNCs compared with the initial pNC solution. Thus, the use of TEOS molecules or silica matrices together with proper separation of pNCs is believed to preserve the optical properties of initial nanoparticles. Indeed, the mild heating of pNCs with TEOS molecules resulted in a less pronounced redshift of the PL band (from 518 to 520 nm) with the same absorption spectrum compared to initial pNCs (Figure 5a,b). The FWHM slightly increased from 19 to 21 nm compared to bare pNCs. It is worth mentioning that the pNC/TEOS sample was very stable while storing at ambient conditions and even when dispersed in polar media such as acetone or water (Figure S4, Supplementary Materials).

The PL spectra of composites are shown in Figure 5c. The PL peak position is blueshifted by 10 and 3 nm for pNC@MS and pNC@MS/HCl, respectively, and broadened up to 32 nm (pNC@MS). PL peak position for pNC@MS/TEOS is redshifted to 522 nm with FWHM of 21 nm. Composites based on pNCs with TEOS molecules embedded in MSs and MS/HCl also showed slight changes in the PL spectra compared to the initial pNC solution (Figure S5, Supplementary Materials). The PL decays for all samples shown in Figure 5d were fitted by biexponential function. The pNC and pNC/TEOS drop cast on the glass slide show an almost similar average PL lifetime of 6.9 and 5.1 ns, respectively. For the composite materials the average PL lifetime is 6.4, 24.1, and 4.3 ns for pNC@MS, pNC@MS/TEOS, and pNC@MS/HCl, respectively. The embedding of pNCs into MSs does not result in the change of the number of nonradiative channels with a weak effect on the steady-state optical properties, thus this matrix is suitable for the further development of composite materials based on pNCs. At the same time, the interface between the pNCs and MSs plays a great role in the bonding formation between pNCs and matrix together with the effect on their radiative transitions. The HCl-treatment of MSs results in the weaker penetration of pNCs inside the MSs together with increased nonradiative channels of the charge recombination for pNCs. In contrast, the pNC@MS/TEOS shows better bonding formation at the pNC-MS interface and improved time-resolved luminescent properties with the almost unchanged steady-state emission spectrum. In addition, the monitoring of the PL signal for 3 days storage of the samples showed that the interface between pNCs and MSs with TEOS molecules prevents their degradation compared to that with HCl-treated MS’s surface (Figure S5, Supplementary Materials). Thus, introducing the TEOS molecules at the pNC-matrix interface resulted in preserving the optical responses and improving their stability upon storage in ambient. To confirm the feasibility of using TEOS as a molecule for pNC surface modification, further experiments on the different chemical compositions of pNCs and matrices need to be carried out.

### TEOS-Treated Lead-Free pNCs

**Figure 6:**
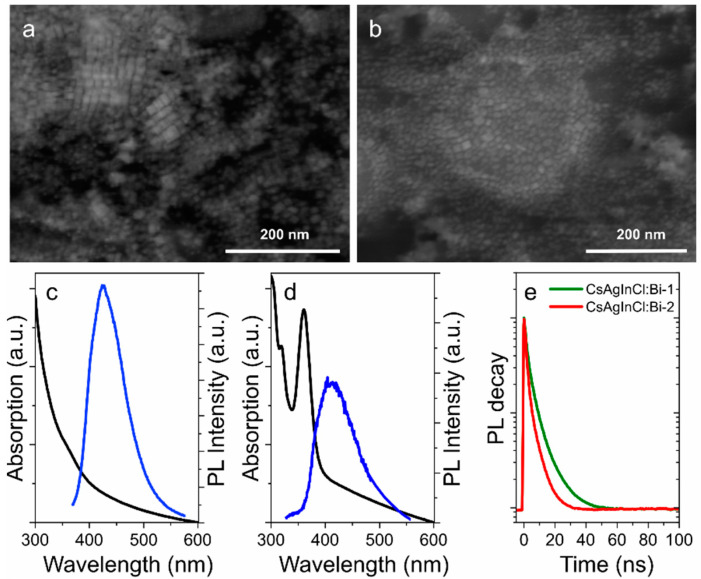
STEM images of CsAgInCl:Bi-1 (**a**) and CsAgInCl:Bi-2; (**b**) pNCs. Absorption spectra (black) and PL spectra (blue) of CsAgInCl:Bi-1; (**c**) and CsAgInCl:Bi-2; (**d**) pNCs in toluene solution. (**e**) PL decay curves.

The success in the improvement of the optical properties of pNC-based composites with the use of TEOS molecules and silica matrix inspired us to implement this approach for the fabrication of lead-free perovskite nanomaterials on the example of Bi-doped Cs_2_AgInCl_6_ double perovskites. Since lead-free perovskite NCs are highly unstable [20], the TEOS molecules were injected during the synthesis. As a result of the synthesis described in Supporting information, CsAgInCl:Bi-1 and CsAgInCl:Bi-2 pNCs were obtained at 280 and 200 °C, respectively, and then covered with TEOS molecules at 80 °C under an inert atmosphere. The crude product was then thoroughly washed to remove the excess of the residual precursors and TEOS molecules. The STEM images are shown in Figure 6. The size of synthesized lead-free pNCs were 13 ± 2 and 12 ± 3 nm for CsAgInCl:Bi-1 and CsAgInCl:Bi-2 pNCs, respectively. From Figure 6, it is seen that pNCs synthesized at 280 °C have cubic shape whereas pNCs synthesized at 200 °C show ellipsoid shape of particles. Since the perovskite materials are characterized by the cubic crystal structure, this observation suggests that the increased temperature results in more crystallized nanoparticles and a decrease of the byproducts from the precursors, such as silver nanoparticles according to Ref. [29]. From STEM images it is hard to confirm the success of the silica layer formation at the pNC surface. The presence of TEOS molecules at the pNC surface was further investigated by FTIR spectra shown in Figure S6 (Supplementary Materials). FTIR spectra of both samples contain peaks at {3080, 2920, 2850} cm^−1^ are typical for –CH stretching, 1712 cm^−1^, 1642 cm^−1^ and 1466 cm^−1^ are attributed to the vibrations of the ligand shell, carboxylic, amine, and alkene groups, respectively. The peaks at {1166, 1103, 1079} and {965, 790} cm^−1^ are similar to that observed for pNC/TEOS sample and are attributed to vibrations of TEOS molecule which confirm the successful silica shell growth at the CsAgInCl:Bi pNC surface. 

The optical properties of synthesized NCs are shown in Figure 6c–e. CsAgInCl:Bi-1 pNCs have monotonously increasing optical density towards blue spectral region with a shoulder at 360 nm. The PL band is centered at 435 nm with FWHM of 95 nm which is relatively broad for the perovskite materials [1]. CsAgInCl:Bi-2 pNCs have more pronounced absorption peaks observed at 318 and 365 nm with a less intense PL band centered at 420 nm and FWHM of 105 nm. The PL band peak position of CsAgInCl:Bi-2 is blueshifted together with increased FWHM value compared to that of CsAgInCl:Bi-1 sample which can be attributed to the morphology of nanoparticles. PL decays were fitted by biexponential function and average PL lifetimes were estimated as 5.2 and 3.5 ns for CsAgInCl:Bi-1 and CsAgInCl:Bi-2, respectively. The PL quantum yield estimated as relative to Rhodamine 6G was of 5.6% and 1.4% for CsAgInCl:Bi-1 and CsAgInCl:Bi-2, respectively. The analysis of optical properties shows that the morphology of pNCs affects their emission: the better the crystallinity of the particles with increased silica layer thickness, the more intense PL signal which is accompanied with increased PL lifetime. It is worth mentioning that the synthesis of lead-free pNCs without TEOS molecules resulted in poor stability of optical responses of pNCs: the emission disappears during the opening of the flask almost immediately. However, both CsAgInCl:Bi-1 and CsAgInCl:Bi-2 TEOS-covered pNCs were stable as dried powders during 6 months storage in the fridge (at 5 °C). 

## 4. Conclusions

We developed the method of formation of composite materials based on pNCs and TEOS molecules and silica MSs which allow control of the interaction between the pNCs and matrix at their interface. The treatment of pNCs, both CsPbBr_3_ and Cs_2_AgInCl_6_:Bi, with TEOS molecules results in the preserved optical properties with increased stability during the storage in ambient conditions. The TEOS molecules also provide the protection against decomposition of pNCs crystal structure while dissolving in polar media. The treatment of the MSs with TEOS molecules followed by the pNCs embedding results in the improved pNCs penetration rate and optical parameters, such as an increase of the average PL lifetime. As was previously shown the use of silica precursors resulted in increased stability of optical responses from 12 h in the air [16] to up to 15 days in water [30] due to efficient passivation and protection of pNC from the environment. Thus, the developed in this work approach to change the chemical composition of the interface of pNC-based composites with TEOS molecules has proved to expand a toolkit in fabrication procedures of composites with improved performance for future photonics.

## Data Availability

The data presented in this study are available on request from the corresponding author.

## Supplementary Materials

The following are available online at https://www.mdpi.com/2079-4991/11/1/119/s1, Materials; synthesis of pNCs; optical properties of composite materials based on pNCs.
