# Supplementary material for: Interface Chemical Modification between All-Inorganic Perovskite Nanocrystals and Porous Silica Microspheres for Composite Materials with Improved Emission
Source: Nanomaterials (Basel). 2021 Jan 7;11(1):119. doi: 10.3390/nano11010119 (PMC7825651; doi:10.3390/nano11010119)
Supplement: Supplementary file 1 [file nanomaterials-11-00119-s001.pdf]

nanomaterials

Supplementary Materials

Interface Chemical Modification between All-Inorganic Perovskite Nanocrystals and Porous Silica Microspheres for Composite Materials with Improved Emission

Sergei Cherevkov [1,†], Ruslan Azizov [1,†], Anastasiia Sokolova [1], Valeriia Nautran [1], Mikhail Miruschenko [1], Irina Arefina [1], Mikhail Baranov [1], Dmitry Kurdyukov [2], Ekaterina Stovpiaga [2], Valery Golubev [2], Alexander Baranov [1] and Elena Ushakova [1,3,*]

1 Center of Information Optical Technologies, ITMO University, 197101, Saint Petersburg, Russia; s.cherevkov@gmail.com (S.C.); azizov.ruslan.2008@mail.ru (R.A.); physasv@yandex.ru (A.S.); l.nautran@mail.ru (V.N.); miruschenko98mail@yandex.ru (M.M.); irina-arefina97@mail.ru (I.A.); mbaranov@mail.ru (M.B.); a_v_baranov@yahoo.com (A.B.)

2 Laboratory of Amorphous Semiconductor Physics, Ioffe Institute, 194021, Saint Petersburg, Russia; kurd.gvg@mail.ioffe.ru (D.K.); kattrof@gvg.ioffe.ru (E.S.); golubev.gvg@mail.ioffe.ru (V.G.)

3 Department of Materials Science and Engineering and Center for Functional Photonics (CFP), City University of Hong Kong, Hong Kong, China

* Correspondence: elena.ushakova@itmo.ru

† S.C. and R.A. contributed equally to this work.

Materials

Chemicals: Cs_2CO_3 (99.9%, Aldrich, Darmstadt, Germany), $AgNO_3$ (99.9%, Aldrich, Darmstadt, Germany), $InCl_3$ (99.99%, Aldrich, Darmstadt, Germany), $BiCl_3$ (99.99%, Aldrich, Darmstadt, Germany), $PbBr_2$ (99.999%, Aldrich, Darmstadt, Germany), 1-octadecene (ODE, 90%, Aldrich, Darmstadt, Germany), oleic acid (OA, 85-92%, Fisher, Loughborough, UK), oleylamine (OLA, 80–90%, Aldrich, Darmstadt, Germany), hexane (95%, Aldrich, Darmstadt, Germany), hydrochloric acid (HCl, analytical pure, Vecton, St Petersburg, Russia), hydrobromic acid (HBr, ≥99.99%, 48 wt% in H_2O, Aldrich, Darmstadt, Germany), N,N-dimethylformamide (DMF, anhydrous, 99.8%, Aldrich, Darmstadt, Germany), and tetraethyl orthosilicate (TEOS, 99.99%, Aldrich, Darmstadt, Germany). Toluene (99.8%) and acetone (99.8%) were bought from Alfa Aesar (Kandel, Germany). All chemicals were used as received without further purification.

Synthesis of Cs–Oleate. 162.8 mg Cs_2CO_3, 2 mL ODE, and 0.5 mL OA were loaded into a 10 mL flask, dried, and degassed under vacuum at 120 °C for 1 h. Then, to obtain a clear solution, the mixture was heated under Ar+ to 150 °C.

Synthesis of $CsPbBr_3$ NCs based on procedure reported in Ref. [1]. To degassed solution of 1.25 mL of ODE, 0.125 mL of OA, 0.125 mL of OlAm were added 10 μL of HBr, and 0.1 mL of CsOA precursor (0.1 M, 0.35 g of Cs_2CO_3 degassed in 20 mL of ODE and 1.25 mL of OA at 150 °C) and after that was swiftly injected 0.8 mL of $PbBr_2$ precursor (0.2 M, 73.5 mg $PbBr_2$ in 1 mL of DMF). After 10 seconds, 20 mL of acetone was swiftly added to quench the reaction (the solution turned turbid, and its color evolved slowly to green). The pNCs were precipitated by centrifugation at 3500 rpm for 5 min and then redispersed in toluene.

Synthesis of $Cs_2AgInCl_6$:1% Bi NCs based on procedure reported in Ref. [2]. In a typical reaction, 61 mg $AgNO_3$, 80 mg $InCl_3$, 1.1 mg $BiCl_3$, 14 mL ODE, 2 mL OA, 1 mL OLA, and 0.28 mL HCl were loaded into a 50 mL three-necked flask and heated to 120 °C for 1 h, and then the temperature was raised to 180–280 °C under Ar+ atmosphere, and 0.8 mL of hot Cs–oleate solution was injected quickly under vigorous stirring. Five seconds after the injection, the mixture was cooled down by a water bath. At 80 °C 0.5 mL TEOS was loaded. A centrifugation process (40 min at 6000 rpm) was performed

to separate the NCs from the crude solution. After that, the crude NCs were washed with acetone several times and redispersed in toluene.

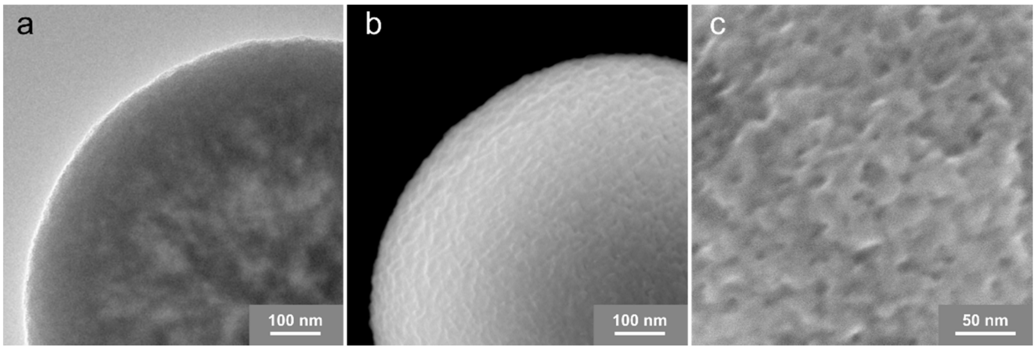

Figure S1. (**a**) STEM and (**b**) SEM image of typical MS. (**c**) SEM of the MS's surface showing porous morphology.

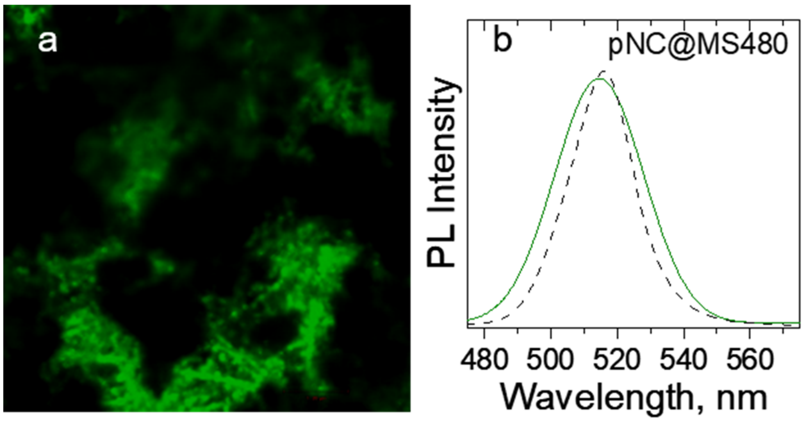

Figure S2. (**a**) PL microscopic image of the pNCs embedded in MSs with diameter of 480 nm. Image size is of 500 μm. (**b**) PL spectrum of composite material compared to that of pNCs in solution.

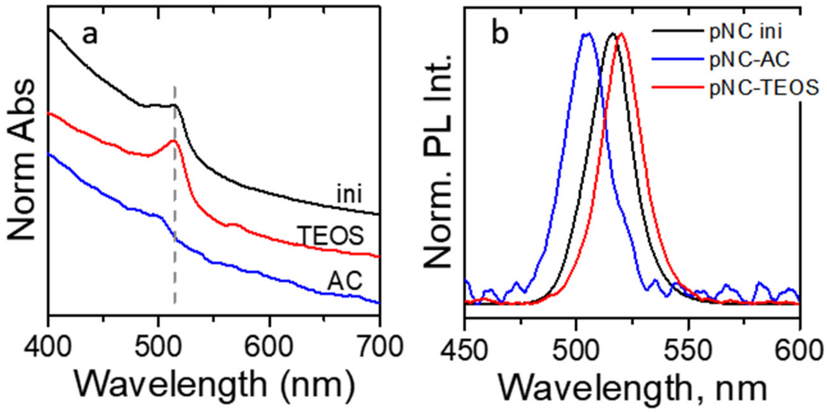

Figure S3. Absorption (**a**) and PL (**b**) spectra of pNC solutions: initial, pNC-ini (black), treated with ammonium chloride, pNC-AC (blue) and TEOS molecules, pNC-TEOS (red).

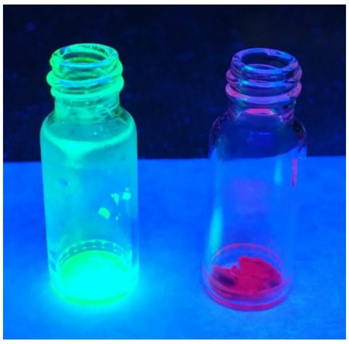

Figure S4. Photo of the samples pNC/TEOS under UV light: green emissive $CsPbBr_3$ (left) and red emissive $CsPb(Br/I)_3$ (right) after storage at ambient conditions for 1 week.

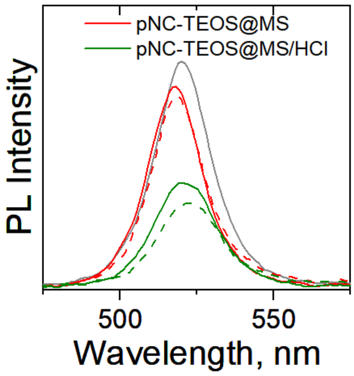

Figure S5. PL spectra of initial solution of pNC with TEOS (grey curve), composites: pNC-TEOS@MS (red curve) and pNC-TEOS@MS/HCl (green curve). By way of dashed lines, PL spectra of samples after 3 days storage at ambient conditions are shown.

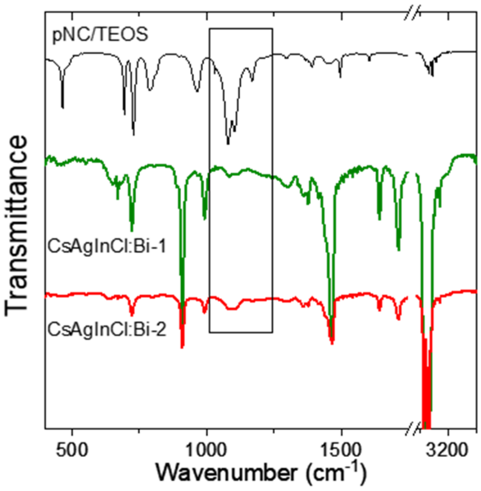

Figure S6. FTIR spectra of CsAgInCl:Bi-1 (green) and CsAgInCl:Bi-2 (red) samples. FTIR spectrum of pNC/TEOS sample (black) is shown for reference. The region attributed to the peaks typical for TEOS molecule is highlighted by rectangle.

References

1. Akkerman, Q.A.; Motti, S.G.; Kandada, A.R.S.; Mosconi, E.; D'Innocenzo, V.; Bertoni, G.; Marras, S.; Kamino, B.A.; Miranda, L.; de Angelis, F., et al. Solution synthesis approach to colloidal cesium lead halide perovskite nanoplatelets with monolayer-level thickness control. *Journal of the American Chemical Society* **2016,** *138.3*, 1010–1016.

2. Liu, Y.; Jing, Y.; Zhao, J.; Liu, Q.; Xia, Z. Design optimization of lead-free perovskite $Cs_2AgInCl_6$: Bi nanocrystals with 11.4% photoluminescence quantum yield. *Chemistry of Materials* **2019**, *31.9*, 3333–3339.

© 2021 by the authors. Licensee MDPI, Basel, Switzerland. This article is an open access article distributed under the terms and conditions of the Creative Commons Attribution (CC BY) license (http://creativecommons.org/licenses/by/4.0/).
